# Analysis of Natural Selection of Immune Genes in *Spinibarbus caldwelli* by Transcriptome Sequencing

**DOI:** 10.3389/fgene.2020.00714

**Published:** 2020-07-24

**Authors:** Yun Tuo, Wuying Chu, Jianshe Zhang, Jia Cheng, Lin Chen, Lingsheng Bao, Tiaoyi Xiao

**Affiliations:** ^1^Hunan Engineering Technology Research Center of Featured Aquatic Resources Utilization, Hunan Agricultural University, Changsha, China; ^2^College of Life Science and Resources Environment, Yichun University, Yichun, China; ^3^Department of Biological and Environmental Engineering, Changsha University, Changsha, China

**Keywords:** *Spinibarbus caldwelli*, transcriptome, natural selection, immune genes, orthologous unigenes

## Abstract

*Spinibarbus caldwelli* is an omnivorous cyprinid fish that is distributed widely in China. To investigate the adaptive evolution of *S. caldwelli*, the muscle transcriptome was sequenced by Illumina HiSeq 4000 platform. A total of 80,447,367 reads were generated by next-generation sequencing. Also, 211,386 unigenes were obtained by *de novo* assembly. Additionally, we calculated that the divergence time between *S. caldwelli* and *Sinocyclocheilus grahami* is 23.14 million years ago (Mya). And both of them diverged from *Ctenopharyngodon idellus* 46.95 Mya. Furthermore, 38 positive genes were identified by calculating Ka/Ks ratios from 9225 orthologs. Among them, several immune-related genes were identified as positively selected, such as *POLR3B*, *PIK3C3*, *TOPORS*, *FASTKD3*, *CYPLP1A1*, and *UACA*. Our results throw light on the nature of the natural selection of *S. caldwelli* and contribute to future immunological and transcriptome studies.

## Introduction

Since the discovery of nucleotide sequence variations (Aquadro and Greenberg, 1983), the question of how adaptive natural selection affects the genome, directly or indirectly, has received wide attention. One of the most significant effects of adaptive natural selection is the impact at the polymorphic level and recombination rate (Corbettdetig et al., 2015). As reported previously, levels of polymorphism and recombination decrease proportionally with respect to increase of natural selection forces (Kaplan et al., 1989; Barton, 1998). Furthermore, there might be different kinds of mutations, as single nucleotide substitution was not the only kind found associated with adaptive selection, such as the number of gene copies (Schrider et al., 2013), the infix of transposable elements (Gonzalez et al., 2008), and large scale inversions (Stefansson et al., 2005; Kirkpatrick and Kern, 2012).

After decades of research, natural selection is an adaptive response to long-term environmental changes (Messer and Petrov, 2013). Fortunately, taking advantage of high-throughput sequencing technology, positively selected genes can be quickly identified from abundant homologous genes. Next-generation sequencing (RNA-seq) has been developed from high-throughput technology, which can supply genetic data without background information of a species. Even if there is no reference genome, the high-quality sequences can still be obtained by *de novo* assembly. For example, by comparing transcriptome data, Ma et al. (2016) have analyzed the adaptive evolution of six catfish species from an altitude gradient. Schartl et al. (2013) have studied the adaptability of the platyfish through genome comparison. Both studies obtained large numbers of gene sequences by high-throughput sequencing.

The current perspective is that positive gene selection is usually associated with immunity and reproduction (Jansa et al., 2003). For example, by transcriptome sequencing, several immune genes show evidence for natural selection in *Parachromis managuensis* (Zhong et al., 2018). Global analysis of transcriptome sequences in *Aldrovandia affinis*, a deep-sea fish, has identified a set of genes related to cytoskeletal structures, which means that DNA repair and treatment of genetic information can contribute to adaptation in the deep-sea environment (Lan et al., 2018). Transcriptome analysis in *Danio albolineatus* and *Danio choprae* has highlighted the accelerated evolution of immune genes, such as *MPG*, *GTF2E1*, *REL*, and *STAT6* in *D. choprae* and *MYCN*, *ADORA2A*, and *CYP17A1* in *D. albolineatus* (Bao and Xia, 2017). These studies stressed that these naturally selected genes were usually related to immunity and resistance in fish. Searching for typical genome changes among species will facilitate the comprehension of biological mechanisms and the genetic basis of adaptation.

As a cultured fish, *S. caldwelli* has brilliant economic prospects, which are attributed to its fast growth and high environmental adaptability. To understand the biogeographical process of *S. caldwelli*, the *CYTB* gene was first employed to identify the biogeographic affinity of *S. caldwelli* from different river basins in China (Tang et al., 2003). Then, the molecular and morphological evidence was used to show that *S. caldwelli* is a valid species (Tang et al., 2005). Genetic polymorphisms have been studied in *Spinibarbus hollandi*, *Spinibarbus sinensis*, and *Spinibarbus denticulatus* by using Random Amplification of Polymorphic DNA (Zhu et al., 2006). Furthermore, Wang et al. (2013) have analyzed the molecular variance and phylogenetic relationships in 61 specimens of *S. caldwelli* based on mitochondrial *CYTB* (Huang et al., 2008). However, studies on the characteristics of adaptive changes at genome level in *S. caldwelli* are rare.

In this study, to find the genes associated with resistance and adaptation, a transcriptome sequencing approach was applied to *S. caldwelli*. The Gene Ontology (GO), Kyoto Encyclopedia of Genes and Genomes (KEGG), and Swiss-Prot databases were employed for function annotation and phylogenetic analysis provided evidence for natural selection. Orthologs were obtained by comparing the genomic data of *S. caldwelli* and six other species. Then, genes under positive selection were identified from the numerous orthologous genes using a branch-site model. The results offered new evidence for positive selection and environmental adaptation in *S. caldwelli* at the genomic level.

## Materials and Methods

### Sample Preparation and Transcriptome Sequencing

The *S. caldwelli* used for experiments were captured from the Jinjiang River and collected from a local market in Yichun, Jiangxi Province, China. Dorsal muscles of fish were sampled from three individual fishes. The tissue samples were quickly frozen by liquid nitrogen and stored at −80°C. All experiments were carried out following the Animal Ethics Committee of Hunan Agricultural University.

Total RNA was extracted following the instruction of RNAiso Plus (Takara Bio Inc., Shiga, Japan). RNA purity was measured using OD_260__/__280_ values using Nanodrop 2000 (Thermo Fisher Scientific Inc., Waltham, MA, United States). The integrity of RNA was assessed using an Agilent 2100 (Agilent Technologies, Inc., Santa Clara, CA, United States). A cDNA library was generated using NEBnext Ultra RNA library prep kit (New England Biolabs, Inc., Ipswich, MA, United States). mRNA transcriptions were enriched by Magnetic Beads Oligo (dT) and sectioned using fragmentation buffer (Life Technologies, Inc., Carlsbad, CA, United States). The first cDNA strand was synthesized using random hexamer primers, and second chain synthesis was performed using DNA polymerase I and RNase H (Life Technologies, Inc.). Subsequently, the outcomes were purified by AMPure XP system (Beckham Coulter, Inc., Brea, CA, United States) and amplified by PCR to build a sequence library. At last, a Bioanalyzer 2100 system (Agilent Technologies, Inc.) was used to assess the quality of the samples and dilute them to a 1.5 μg/mL concentration. The cDNA library was sequenced on a Hiseq 4000 platform (Illumina Inc., San Diego, CA, United States) by the paired-end reads method.

### Quality Control, Raw Read Filtering, and *de novo* Transcriptome Assembly

The raw reads were assessed using CASAVA (v1.8, Illumina Inc.) and filtered to produce clean reads. First, the adaptors were removed. Second, the reads with an N ratio of more than 10% were removed. Finally, low-quality reads (Q_phred_ value of <20 and >50% bases) were trimmed, with a Q_phred_ cut-off value of −10 log_10_(e). Short reads obtained by sequencing were assembled using Trinity software (Grabherr et al., 2011) and the unigenes used for subsequent analysis. TransDecoder was used to identify candidate coding regions based on the following criteria: (1) An ORF that meets the minimum limit length (200 bp) can be found in the transcript sequence. (2) The logarithm likelihood score was greater than 0. (3) The logarithm likelihood score of the first open reading frame (ORF) is the maximum compared with the other 5. (4) If the candidate ORF was completely involved in other ORFs, we only report the longest ORF.

### Gene Prediction

The unigenes and contigs were annotated in several public databases, including Swiss-Prot, KEGG, and GO. Swiss-Prot and KEGG annotation was performed using BLAST 2.2.28 + software (Camacho et al., 2009) with E-values of le-5 and le-10, respectively. GO annotation was performed using the BlAST2GO v2.5 software and the E-value threshold at le-6 (Conesa and Gotz, 2008).

The coding DNA sequence (CDS) of the unigenes was picked out using TransDecoder software. The unigenes noted by Swiss-Prot were translated after CDS extraction. Then, the obtained ORFs were used to search for orthologous sequences.

### Gene Family and Phylogenetic Analyses

After filtering out alternatively spliced transcripts of each gene, only the longest transcripts were retained. The orthologous gene families were identified using the OrthoMCL (v2.0.9) pipeline (Conesa and Gotz, 2008) between *S. caldwelli* and six other species (*Astyanax mexicanus, Danio rerio, Ctenopharyngodon idellus, Latimeria chalumnae, Xiphophorus maculatus*, and *Sinocyclocheilus grahami*). Firstly, we screened the longest transcripts of each gene and then performed BLASTp alignment of all protein sequences of the selected species (E-value ≤ 1e−5). Lastly, the homologous gene families were obtained using the Markov clustering (MCL) algorithm.

After gene family clustering, single-copy genes were used for phylogenetic analysis. Firstly, sequence matrices were initially aligned with MAFFT 7.0 (Katoh and Standley, 2013), then the amino acid sequence was converted to CDS and refined using Gblocks (Gutiérrez-Larruscain et al., 2018). Finally, the GTRGAMMA model was employed to analyze the data with raxml method. Bootstrap was set to 100 and *L. chalumnae* was used as the outgroup specie.

We estimated divergence time with the Bayesian method MCMCTree in PAML v4.9e (Yang, 2007). Fourfold degenerate positions were extracted by CDS sequence alignment of 4126 single-copy orthologous genes that are shared by *S. caldwelli* and other fishes. The parameter of MCMCTREE was set as follows: clock = 2, RootAge ≤ 434.42, model = 7, BDparas = 110, kappa_gamma = 62, alpha_gamma = 11, rgene_gamma = 23.18, sigma2_gamma = 11.3. And the divergence times of *C. idellus* [36.15–64.69 million years ago (Mya)] (Li et al., 2013; Wang et al., 2013), *X. maculatus* (210.16–256.54 Mya) (Betancur-R et al., 2013; Chen et al., 2013), and *L. chalumnae* (424.83–445.83 Mya) (Nakatani et al., 2011; Betancur-R et al., 2015) were added as fossil calibrations.

### Identification of Orthologous Sequences and Detection of Natural Selection

According to the high similarity of homologous genes, a BLASTp search was employed to align the protein sequences of these seven species (E-value ≤ 1e−5) to identify orthologous sequences.

The detection of natural selection was performed using CODEML software in the PAML package using the branch-site model (Zhang et al., 2005). A Chi-square test was employed to verify the significance of obtained positively selected genes. Genes under positive selection were assigned to Swiss-Prot and KEGG categories to identify their related functions.

## Results

### Transcriptome Sequencing and Assembly

**FIGURE 1:**
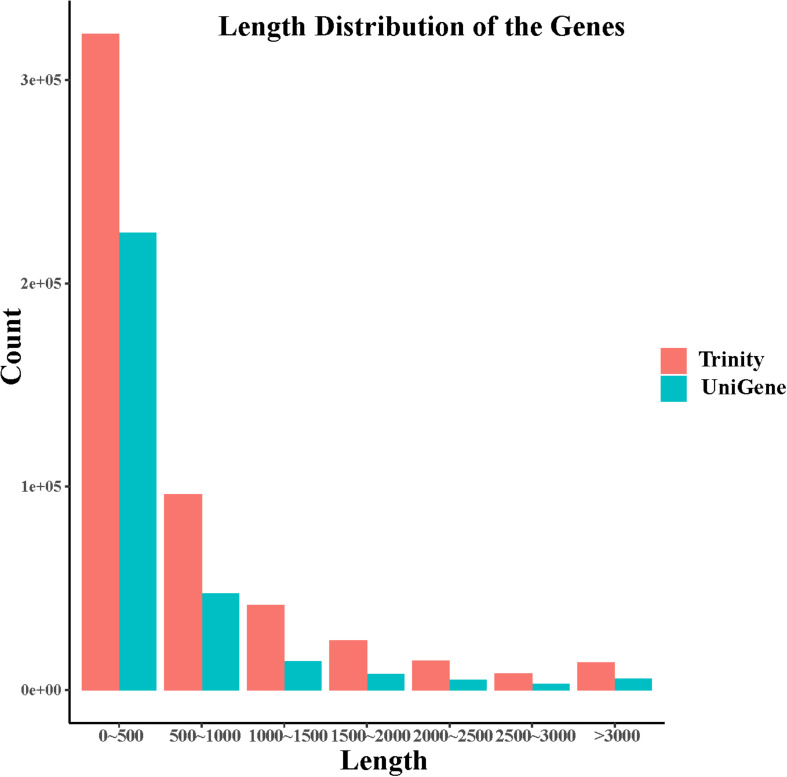
Length distribution of contigs and unigenes derived from *S. caldwelli*.

In this study, after removing the low-quality reads, 80,447,367 reads with 22.41 Gb of clean bases were ultimately obtained. By *de novo* assembly of these clean reads, 387,437 contigs, with N50 of 1077 bp and the average length of 620 bp, were generated. Furthermore, 211,386 unigenes with N50 of 706 bp and the average length of 548 bp have been assembled. contig and unigene data are outlined in Supplementary Table S1 and their length distribution is shown in Figure 1. All raw data can be downloaded in the NCBI Sequence Read Archive database (Accession No. PRJNA563944).

### Gene Prediction

A total of 63,755 ORFs were identified by TransDecoder (Supplementary Table S2). Also, 61,337 unigenes (30.16% of total unigenes) from *S. caldwelli* were annotated in three public databases (Swiss-Prot, GO, and KEGG; Table 1).

**TABLE 1:** Summary of transcriptome assembly from *S. Caldwelli*.

Assembly	
The count of clean reads	80,447,367
The count of unigenes	211,386
N50 length of unigenes (bp)	706
Mean length of unigenes (bp)	548
**Predictions**	
Predicted in Swiss-Prot (counts)	47,843
Predicted in GO (counts)	34,827
Predicted in KEGG (counts)	40,835
Predicted at least in one database (counts)	61,337

**FIGURE 2:**
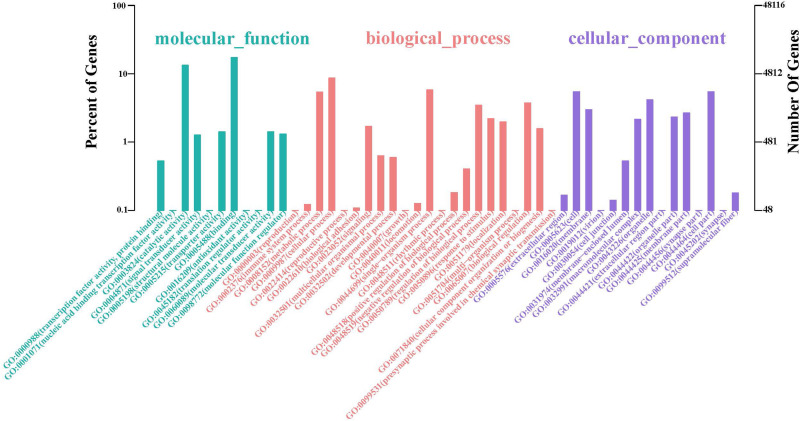
Annotation of unigenes of *S. caldwelli* from the GO database.

The GO database illustrated the experimental results regarding molecular functions, biological processes, and cellular components (Figure 2 and Supplementary Table S3). The top two GO terms in the molecular function hierarchy were catalytic activity and binding, which included 8338 and 6403 unigenes, respectively. Among the biological process hierarchy, cellular, single-organism, and metabolic processes represented the majority of the unigenes (4169, 2788, and 2602, respectively). Besides, in the cellular component hierarchy, the GO terms “cell,” “cell part,” and “organelle” contained the most abundant unigenes (2638, 2638, and 1998, respectively). Additionally, more genes can be found per orthogroup in *S. caldwelli* than in others, and this might be an overestimation issue by Trinity, which is often reluctant to combine multiple isoforms (Cabau et al., 2017).

**FIGURE 3:**
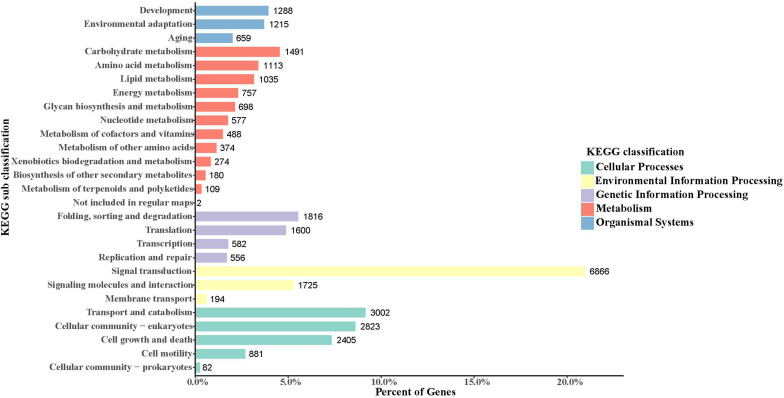
Annotation of unigenes of *S. caldwelli* in the KEGG pathway database. From top to bottom by color: organismal systems, blue; metabolism, red; genetic information processing, purple; environmental information processing, yellow; cellular processes, green.

In the obtained *S. caldwelli* transcriptome, the KEGG pathway database annotated and sorted the unigene functions into five categories, including cellular process pathways, environmental information processing, genetic information processing, metabolism, and organismal systems (9193, 8785, 4544, 7098, and 3162 unigenes, respectively; Figure 3). In the KEGG sub-classification, the categories of signal transduction; transport and catabolism; cellular community-eukaryotes; cell growth and death; and folding, sorting, and degradation (6866, 3002, 2823, 2405, and 1816 unigenes, respectively) were the five major pathways.

### Gene Family and Divergence Time

**FIGURE 4:**
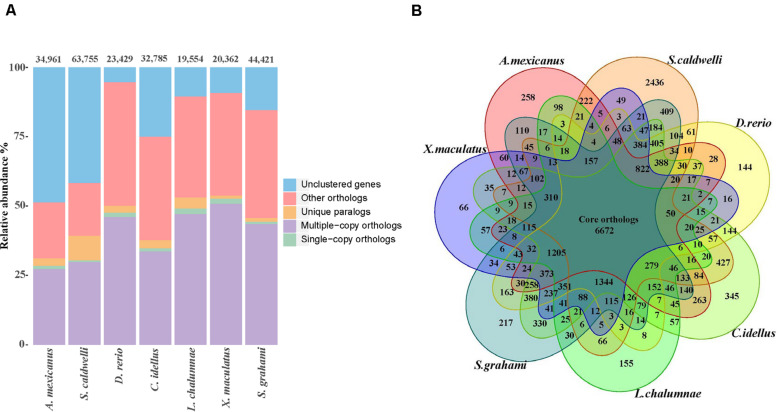
**(A)** The number of genes in the seven fish species, displaying the great quantity of *S. caldwelli* genes in comparison with*S. grahami* and others. The count of multiple copy orthologs from *S. caldwelli* was high. **(B)** Comparison of orthologous genes of *S. caldwelli* and six fish species by Venn diagram.

Through analyses of gene families between *S. caldwelli* and six species, 16,132 gene families containing 37,187 genes were clustered. Among them, 2436 gene families were unique in *S. caldwelli* (Figure 4A). Furthermore, *S. caldwelli* and *S. grahami* had the largest number of shared gene families (12,427 gene families) among these species of fish (Figure 4B).

**FIGURE 5:**
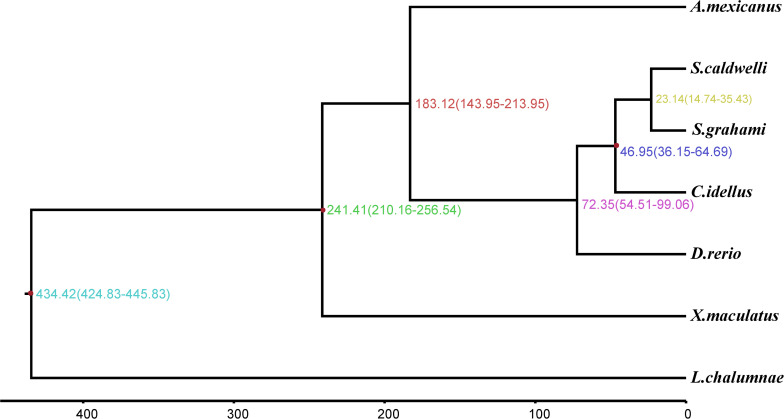
The phylogenetic tree and molecular timing of *S. caldwelli* and other fish species, showing *S. caldwelli* had diverged 23.4 million years ago. A total of 13,568 fourfold degenerate positions are used to compute the tree and infer the divergence dates.

According to the molecular clock theory, the codons are replaced with each other in an almost constant ratio over time. But the encoded amino acid does not change with the substitution of the third base of the fourfold degenerate synonymous sites (neutral evolution) (Kimura, 1983). Therefore, the fourfold degenerate synonymous sites in single-copy gene families were usually used to estimate the divergence time between species. In this study, we calculated that the divergence time between *S. caldwelli* and *S. grahami* is 23.14 Mya. And both of them diverged from *C. idellus* 46.95 Mya (Figure 5).

### Orthologic Identification and Accelerated Evolution Gene Detection

To further understand the evolutionary dynamics of *S. caldwelli*, BLASTp (E-value ≤ 1e−5) searching among *S. caldwelli* and other six species identified a total of 9225 putative orthologs.

Moreover, by the branch-site model, 38 positively selected genes were found and all of them annotated in the Swiss-Prot database (Supplementary Table S4).

**FIGURE 6:**
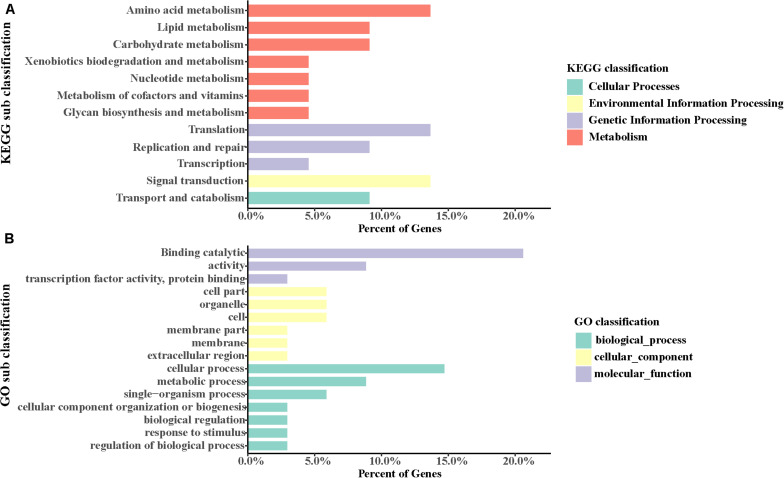
**(A,B)** Positive gene assignments in GO and KEGG pathway, respectively.

GO categories were employed to annotate these genes. It was found that the most abundant GO terms of positively selected genes in *S. caldwelli* were “cellular process” and “binding.” Also, there were positively selected genes identified by the term “response to stimulus” (Figure 6A).

Also, using the KEGG pathway database, some genes were identified that might have accelerated evolution. The most two abundant pathways with accelerated evolution genes were “amino acid metabolism” and “translation” (Figure 6B).

The InnateDB database Breuer et al. (2013) was employed to identify the positively selected genes associated with the immune system. Consequently, six immune system genes were found, including DNA-directed RNA polymerase III subunit RPC2 (*POLR3B*), phosphatidylinositol 3-kinase catalytic subunit type 3 (*PIK3C3*), E3 ubiquitin-protein ligase Topors (*TOPORS*), fast kinase domain-containing protein 3 (*FASTKD3*), cytochrome P450 1A1 (*CYP1A1*), and Uveal autoantigen with coiled-coil domains and ankyrin repeats protein (*UACA*) (Table 2).

**TABLE 2:** Positively selected genes associated with immune from InnateDB.

Orthologs	Gene name	Symbol	*p*-Value	Function
ortholog07321	DNA-directed RNA polymerase III subunit RPC2	*POLR3B*	2.22E-16	Metal ion binding
ortholog03076	Phosphatidylinositol 3-kinase catalytic subunit type 3	*PIK3C3*	2.16E-13	Transferase activity
ortholog19412	E3 ubiquitin-protein ligase Topors	*TOPORS*	1.80E-04	Metal ion binding
ortholog07361	Fast kinase domain-containing protein 3	*FASTKD3*	8.45E-04	Protein binding
ortholog10495	Cytochrome P450 1A1	*CYP1A1*	4.00E-03	Oxidoreductase activity
ortholog11426	Uveal autoantigen with coiled-coil domains and ankyrin repeats protein	*UACA*	4.96E-03	Protein binding

## Discussion

*Spinibarbus caldwelli* is a medium to large-sized fish, which belongs to the order Cypriniformes, family Cyprinidae, and genus *Spinibarbus* and is widely distributed in the rivers of southern China (Tang et al., 2003). Previous studies on the genetic information of *S. caldwelli* have been mainly based on a few genes. For example, the *CYTB* gene was first employed to identify the biogeographic affinity of *S. caldwelli* from different river basins in China. Then, the molecular and morphological evidence was used to show that *S. caldwelli* is a valid species (Tang et al., 2005). Furthermore, Huang et al. (2008) have analyzed the mitochondrial DNA diversity of *S. caldwelli*. They tested the molecular variance and genetic diversity among 61 specimens of *S. caldwelli* by using mitochondrial *CYTB*. The results showed that the diversity in *S. caldwelli* was lower than other cyprinids (Huang et al., 2008). In this study, the molecular-clock approach predicted divergence between *S. caldwelli* and *S. grahami* is 23.14 Mya with confidence interval 14.74–35.43. Both of them diverged from *C. idellus* 46.95 Mya with confidence interval 36.15–64.69. These results were consistent with previous studies and further refined the speciation time of *S. caldwelli*.

Previous studies of various species, such as fish (Xiao et al., 2015), insects (Roux et al., 2014), and bats (Hawkins et al., 2019), have suggested that positively selected genes can be related to immune function (Elmer et al., 2010), energy supplying (Ma et al., 2016), collagen production (Hawkins et al., 2019), DNA repair (Lan et al., 2018), and response to hypoxia (Ma et al., 2016). Ren et al. (2014) found *GATA-binding protein 4* (*GATA4*), Transducin (beta)-like 3 (*TBL3*), PHD finger protein 13 (*PHF13*), and Neutral cholesterol ester hydrolase 1 (*NCEH1*) in *Erythroculter ilishaeformis* exhibit strong positive selection metabolic processes and development. Zou et al. (2014) compared the transcriptome of *Elopichthys bambusa* with *Hypophthalmichthys molitrix* and *Megalobrama amblycephala*, revealing multiple candidate adaptive genes in each species, such as zona pellucida glycoprotein 2 in *E. bambusa* and zebrafish vitelline envelope protein in *M. amblycephala*. In *Gymnocypris selincuoensis*, zona pellucida sperm binding protein 3 (*Zp3*) and homeobox transcription factor Nanog (*Nanog*) associated with reproduction may be involved in adaption to the strong ultraviolet (UV) radiation (Feng et al., 2019). The genome-wide study on *Gymnocypris przewalskii* showed that positive selection on genes involved in energy metabolism, immune system, and hypoxia response contributed to highland adaption (Tong et al., 2017). Meanwhile, 138 positively selected genes were detected in a deep-sea fish (*A. affinis*), associating with cytoskeleton structures, DNA repair, and genetic information processing (Lan et al., 2018).

There are plentiful studies focusing on the positive selection of immune-related genes. To illustrate, by transcriptome sequencing, Zhong et al. (2018) screened 105 positively selected genes in *P. managuensis*. Among them, the genes encoding for phosphoinositide 3-kinase adapter protein 1, Ras-related protein Rap-1b, complement factor I, and probable ATP-dependent RNA helicase DHX58 were related to immune adaptation. Li et al. (2019) found that B cell-mediated immunity, chemokine-mediated signaling pathway, and immunoglobulin mediated immune response were the positively selected biological processes in big head carp, whereas those in silver carp mainly included the antigen processing and presentation, defense response to fungus, and detection of bacteria. Chen et al. (2008) revealed that TLR9 gene was positive selection in teleosts. Xiao et al. (2015) gave evidence that several immune-related genes, such as *Notch2* and *Nfatc3b*, were identified as positively selected genes in tilapia. The study in *Leuciscus waleckii* also showed that most of the genes were associated with stress adaption and immunity among 61 positive selection genes (Xu et al., 2013). Xu et al. (2016) identified 40 full-length miiuy croaker MHC class IIA (Mimi-DAA) functional alleles from 26 miiuy croaker individuals and found 22 positively selected sites on the Mimi-DAA alleles. Among them, five sites were related to the binding of peptide antigen, indicating that these selected residues may play a crucial role in immune function (Xu et al., 2016).

To identify the potential genes involved in natural selection, the positively selected genes were screened. A total of 38 positively selected genes were identified (Figure 6). The most abundant KEGG pathways with positively selected genes included “amino acid metabolism,” “translation,” and “signal transduction.” These genes were assigned to GO categories subsequently. It has been indicated by the analysis that several positively selected loci or genes were found in multiple biological process in *S. caldwelli*. Therefore “binding catalytic” and “cellular process” were the most abundant GO terms associated with positively selected genes. Meanwhile, “response to stimulus” was also found with positively selected genes in *S. caldwelli* (Figure 6B).

By searching in the InnateDB database, several candidate genes associated with the immune system were found in *S. caldwelli*, including *POLR3B*, *PIK3C3*, *TOPORS*, *FASTKD3*, *CYP1A1*, and *UACA*. *POLR3B* is an antiviral molecule, which has been reported capable of confusing purine metabolism and has something to do with Alzheimer’s disease (Ansoleaga et al., 2015). A study on the relationship of brain immune genes with social behavior in strains of inbreeding rat shows that *POLR3B* is connected to behavioral processes that are essential to their social activities (Ma et al., 2015). Yee et al. (2007) reported that the causative mutation of *POLR3B* can jeopardize digestive organ development in zebrafish. *PIK3C3* is an enzyme that generates phosphatidylinositol 3-phosphate (PI3P), a membrane component, in zebrafish. Lack of *PIK3C3* causes intestinal inflammation and injury (Zhao et al., 2018). Kariuki et al. (2010) have found that the *PIK3C3* locus is related to morbidity from systemic lupus erythematosus (SLE). *TOPORS* is a topoisomerase-I and p53-binding protein that is dynamically related to PML nuclear bodies (Rajendra et al., 2004). It has been predicted that *TOPORS* is a tumor suppressor in colon cancer and other malignancies (Rajendra et al., 2004). Besides, Chakarova et al. (2010) noted that *TOPORS* regulates retinal development in zebrafish. *FASTKD3*, a necessary component of mitochondrial respiration, belongs to the FAST kinase domain-containing protein (FASTKD) family, and therefore has a relationship with cell energy modulation (Simarro et al., 2010). *CYP1A1* activates polycyclic aromatic hydrocarbons, which are turned into reactive intermediates associated with toxicity and carcinogenesis (Shigeyuki et al., 2006). It also plays an important role in detoxification (Shigeyuki et al., 2006). Interestingly, *CYP1A1* and *CYP17A1* previously reported in *D. albolineatus* (Bao and Xia, 2017) are both related to Cytochrome P450, arranged by substrate type pathway. *UACA* has been considered to be a potential target autoantigen in Vogt-Koyanagi-Harada, Behçet’s disease, and sarcoidosis, consequently leading to panuveitis (Yamada et al., 2001). Interestingly, despite that several immune-related genes have been found under selection, our dataset failed to identify other loci that are usually under selection in fish related to the immune system like *MhC*. This may be attributed to difficulty among detection in the presence of the reduced expression of these (genes) loci when using muscular tissue. It may also point to some specificity of the muscular immune system.

## Conclusion

In this article, the muscle transcriptome of *S. caldwelli* was obtained through *de novo* assembly of its transcriptome and screening for accelerated evolution genes using the branch-site model. Based on this data, we calculated that the divergence time between *S. caldwelli* and *S. grahami* is 23.14 Mya. And both of them diverged from *C. idellus* 46.95 Mya. Thirty-eight positively selected loci and genes were identified in *S. caldwelli*. Several genes were found related to immune functions. These results demonstrated the accelerated genetic evolution of these immune genes. The present results provided new insight into evolutionary pressures and significant in the understanding of function and immunology of *S. caldwelli.*

## Data Availability Statement

The sequencing data in our article has been uploaded to the NCBI Sequence Read Archive database (SRR10123035).

## Ethics Statement

The animal study was reviewed and approved by the Animal Care and Use Committee (IACUC) of Hunan Agricultural University (File code: ACC2017007).

## Author Contributions

TX and JZ: methodology. LB: software. YT: validation, writing—original draft preparation, and writing—review and editing. WC: formal analysis and supervision. JC: investigation. TX: resources, project administration, and funding acquisition. LC: data curation. JZ: visualization. All authors contributed to the article and approved the submitted version.

## Conflict of Interest

The authors declare that the research was conducted in the absence of any commercial or financial relationships that could be construed as a potential conflict of interest.
