# Supplementary material for: Analysis of Natural Selection of Immune Genes in Spinibarbus caldwelli by Transcriptome Sequencing
Source: Front Genet. 2020 Jul 24;11:714. doi: 10.3389/fgene.2020.00714 (PMC7393255; doi:10.3389/fgene.2020.00714)
Supplement: Supplementary file 1 [file Data_Sheet_1.docx]

Table S1 The data of RNA-seq from *S. caldwelli*

| **Sample** | **Total**  **length** | **Total**  **genes** | **A verage length** | **Median length** | **N50**  **length** | **Max**  **length** | **Min**  **length** |
| --- | --- | --- | --- | --- | --- | --- | --- |
| UniGene | 115906253 | 211386 | 548.32 | 321 | 706 | 18938 | 201 |
| Trinity | 260635891 | 387437 | 672.72 | 362 | 1077 | 18938 | 201 |

Table S2 The CDS of RNA-seq from *S. caldwelli*

| **Sample** | **Total**  **length** | **Total**  **genes** | **A verage length** | **Median length** | **N50**  **length** | **Max**  **length** | **Min**  **length** |
| --- | --- | --- | --- | --- | --- | --- | --- |
| CDS | 56807976 | 63755 | 891.04 | 567 | 1224 | 26826 | 255 |
| Protein | 18935992 | 63755 | 297.01 | 189 | 408 | 8942 | 85 |

|  |  |  |  |  |  |  |
| --- | --- | --- | --- | --- | --- | --- |
|  |  |  |  |  |  |  |
|  |  |  |  |  |  |  |

Table S3 Summary of GO classification statistics from *S. caldwelli*

| GO database | GO terms | Number of genes |
| --- | --- | --- |
| Molecular function | GO:0000988(transcription factor activity, protein binding) | 254 |
| Molecular function | GO:0001071(nucleic acid binding transcription factor activity) | 38 |
| Molecular function | GO:0003824(catalytic activity) | 6403 |
| Molecular function | GO:0004871(signal transducer activity) | 609 |
| Molecular function | GO:0005198(structural molecule activity) | 29 |
| Molecular function | GO:0005215(transporter activity) | 679 |
| Molecular function | GO:0005488(binding) | 8338 |
| Molecular function | GO:0016209(antioxidant activity) | 16 |
| Molecular function | GO:0045182(translation regulator activity) | 5 |
| Molecular function | GO:0060089(molecular transducer activity) | 679 |
| Molecular function | GO:0098772(molecular function regulator) | 629 |
| Cellular component | GO:0005576(extracellular region) | 80 |
| Cellular component | GO:0005623(cell) | 2638 |
| Cellular component | GO:0016020(membrane) | 1431 |
| Cellular component | GO:0019012(virion) | 2 |
| Cellular component | GO:0030054(cell junction) | 68 |
| Cellular component | GO:0031974(membrane-enclosed lumen) | 254 |
| Cellular component | GO:0032991(macromolecular complex) | 1034 |
| Cellular component | GO:0043226(organelle) | 1998 |
| Cellular component | GO:0044421(extracellular region part) | 9 |
| Cellular component | GO:0044422(organelle part) | 1119 |
| Cellular component | GO:0044425(membrane part) | 1286 |
| Cellular component | GO:0044456(synapse part) | 8 |
| Cellular component | GO:0044464(cell part) | 2638 |
| Cellular component | GO:0045202(synapse) | 8 |
| Cellular component | GO:0099512(supramolecular fiber) | 87 |
| Cellular component | GO:0000003(reproduction) | 34 |
| Cellular component | GO:0002376(immune system process) | 59 |
| Cellular component | GO:0008152(metabolic process) | 2602 |
| Cellular component | GO:0009987(cellular process) | 4169 |
| Biological process | GO:0022414(reproductive process) | 34 |
| Biological process | GO:0022610(biological adhesion) | 52 |
| Biological process | GO:0023052(signaling) | 814 |
| Biological process | GO:0032501(multicellular organismal process) | 303 |
| Biological process | GO:0032502(developmental process) | 287 |
| Biological process | GO:0040007(growth) | 12 |
| Biological process | GO:0040011(locomotion) | 61 |
| Biological process | GO:0044699(single-organism process) | 2788 |
| Biological process | GO:0048511(rhythmic process) | 11 |
| Biological process | GO:0048518(positive regulation of biological process) | 88 |
| Biological process | GO:0048519(negative regulation of biological process) | 194 |
| Biological process | GO:0050789(regulation of biological process) | 1663 |
| Biological process | GO:0050896(response to stimulus) | 1058 |
| Biological process | GO:0051179(localization) | 951 |
| Biological process | GO:0051704(multi-organism process) | 32 |
| Biological process | GO:0065007(biological regulation) | 1802 |
| Biological process | GO:0071840(cellular component organization or biogenesis) | 756 |
| Biological process | GO:0099531(presynaptic process involved in chemical synaptic transmission) | 7 |

Table S4 List of positively selected genes annotated by Swiss-prot database

| Unigenes ID | Annotation | Symbol | *p-value* | Categories |
| --- | --- | --- | --- | --- |
| ortholog02471 | Fanconi anemia core complex-associated protein 100 | FAAP100 | 7.01E-03 | 30 |
| ortholog04020 | DNA-3-methyladenine glycosylase | MPG | 1.92E-08 | 9 |
| ortholog02219 | Tyrosine--tRNA ligase, mitochondrial | YARS2 | 4.34E-06 | 13 |
| ortholog08386 | Histone-lysine N-methyltransferase SETD7 | SETD7 | 5.68E-08 | 11 |
| ortholog03076 | Phosphatidylinositol 3-kinase catalytic subunit type 3 | PIK3C3 | 2.16E-13 | 3 |
| ortholog15868 | Zinc finger FYVE domain-containing protein 26 | ZFYVE26 | 2.05E-02 | 33 |
| ortholog08758 | Centrosomal protein of 104 kDa | CEP104 | 2.12E-08 | 10 |
| ortholog06274 | Glycosylphosphatidylinositol anchor attachment 1 protein | GPAA1 | 2.04E-04 | 19 |
| ortholog00821 | Ganglioside-induced differentiation-associated protein 1 | GDAP1 | 9.89E-11 | 7 |
| ortholog07321 | DNA-directed RNA polymerase III subunit RPC2 | POLR3B | 2.22E-16 | 1 |
| ortholog19412 | E3 ubiquitin-protein ligase Topors | TOPORS | 1.80E-04 | 17 |
| ortholog00333 | Glucocorticoid modulatory element-binding protein 2 | GMEB2 | 2.61E-04 | 20 |
| ortholog22202 | Threonine synthase-like 2 | THNSL2 | 2.08E-02 | 34 |
| ortholog07361 | FAST kinase domain-containing protein 3 | FASTKD3 | 8.45E-04 | 22 |
| ortholog10495 | Cytochrome P450 1A1 | CYP1A1 | 4.00E-03 | 25 |
| ortholog10153 | Golgin subfamily A member 1 | GOLGA1 | 1.73E-02 | 32 |
| ortholog03666 | Williams-Beuren syndrome chromosomal region 16 protein | WBSCR16 | 0 | 36 |
| ortholog00483 | WD repeat-containing protein 41 | WDR41 | 7.46E-03 | 31 |
| ortholog09490 | Protein KIAA0100 | KIAA0100 | 1.84E-04 | 18 |
| ortholog07206 | Maspardin | SPG21 | 5.42E-12 | 5 |
| ortholog04973 | Galactosylceramide sulfotransferase | GAL3ST1 | 3.28E-09 | 8 |
| ortholog03909 | Nucleolin | NCL | 7.75E-05 | 16 |
| ortholog18585 | Epithelial splicing regulatory protein 2 | ESRP2 | 7.52E-13 | 4 |
| ortholog01226 | UPF0696 protein C11orf68 homolog | P5436 | 2.82E-03 | 24 |
| ortholog11426 | Uveal autoantigen with coiled-coil domains and ankyrin repeats protein | UACA | 4.96E-03 | 27 |
| ortholog09425 | Exportin-T | XPOT | 1.23E-05 | 14 |
| ortholog11739 | L-2-hydroxyglutarate dehydrogenase, mitochondrial | L2HGDH | 0 | 36 |
| ortholog07446 | Transmembrane emp24 domain-containing protein 2 (Fragment) | TMED2 | 4.17E-02 | 35 |
| ortholog09996 | Pigment epithelium-derived factor | SERPINF1 | 2.35E-11 | 6 |
| ortholog11559 | Ubiquitin-conjugating enzyme E2 variant 3 | UEVLD | 1.45E-03 | 23 |
| ortholog03317 | HAUS augmin-like complex subunit 4 | HAUS4 | 8.33E-15 | 2 |
| ortholog02562 | Golgin subfamily A member 2 | GOLGA2 | 6.89E-03 | 28 |
| ortholog09325 | Peptide deformylase, mitochondrial | PDF | 3.36E-04 | 21 |
| ortholog04337 | Ubiquitin-like domain-containing CTD phosphatase 1 | UBLCP1 | 8.05E-07 | 12 |
| ortholog04443 | Delta-1-pyrroline-5-carboxylate dehydrogenase, mitochondrial | ALDH4A1 | 0 | 36 |
| ortholog06017 | Transmembrane protein 131 | TMEM131 | 4.67E-03 | 26 |
| ortholog12583 | Nucleolar protein 56 | NOP56 | 3.18E-05 | 15 |
| ortholog15973 | Uncharacterized protein C4orf29 homolog |  | 6.99E-03 | 29 |
